# Treatment of hepatic venous system hemorrhage and carbon dioxide gas embolization during laparoscopic hepatectomy *via* hepatic vein approach

**DOI:** 10.3389/fonc.2022.1060823

**Published:** 2023-01-05

**Authors:** Zhen Qu, Ke-jia Wu, Jia-wei Feng, Ding-sen Shi, Yu-xiang Chen, Dong-lin Sun, Yun-Fei Duan, Jing Chen, Xiao-zhou He

**Affiliations:** The Third Affiliated Hospital of Soochow University, Changzhou First People’s Hospital, Changzhou, China

**Keywords:** laparoscopy, hepatectomy, hepatic vein, hemorrhage, gas embolism

## Abstract

With the improvement of laparoscopic surgery, the feasibility and safety of laparoscopic hepatectomy have been affirmed, but intraoperative hepatic venous system hemorrhage and carbon dioxide gas embolism are the difficulties in laparoscopic hepatectomy. The incidence of preoperative hemorrhage and carbon dioxide gas embolism could be reduced through preoperative imaging evaluation, reasonable liver blood flow blocking method, appropriate liver-breaking device, controlled low-center venous pressure technology, and fine-precision precision operation. In the case of blood vessel rupture bleeding in the liver vein system, after controlling and reducing bleeding, confirm the type and severity of vascular damage in the liver and venous system, take appropriate measures to stop the bleeding quickly and effectively, and, if necessary, transfer the abdominal treatment in time. In addition, to strengthen the understanding, prevention and emergency treatment of severe CO2 gas embolism in laparoscopic hepatectomy is also the key to the success of surgery. This study aims to investigate the methods to deal with hepatic venous system hemorrhage and carbon dioxide gas embolization based on author’s institutional experience and relevant literature. We retrospectively analyzed the data of 60 patients who received laparoscopic anatomical hepatectomy of hepatic vein approach for HCC. For patients with intraoperative complications, corresponding treatments were given to cope with different complications. After the operation, combined with clinical experience and literature, we summarized and discussed the good treatment methods in the face of such situations so that minimize the harm to patients as much as possible.

## Introduction

Liver cancer ranks fifth in cancer incidence and fourth in cancer-related mortality worldwide. And the liver is the sixth primary cancer site (1). Among the many types of liver cancer, HCC occupies the absolute leading number, accounting for 80-90% of primary liver cancer (2).With the development of minimally invasive surgery, laparoscopic hepatectomy (LH) indications expand. The hepatic vein is widely used to guide the approach because of its prominent dissection position (3).Laparoscopic hepatectomy may improve the short - and long-term prognosis after hepatectomy (4). Although there were no significant differences in complication rates or tumor outcomes compared with open hepatectomy, Laparoscopic hepatectomy has the advantages of less blood loss, lower anesthesia dose and shorter hospital stay. Accurate laparoscopic segmentectomy can reduce the abdominal incision and damage to the patient’s body, and it can increase patients’ chances of potential surgery to deal with the recurrence of the disease. Laparoscopic hepatectomy is the best choice under the premise of ensuring the function of the liver and not reducing the curative effect of the operation (5, 6). Therefore, Laparoscopic hepatectomy has been considered the great option for HCC resection in recent EASL guidelines (7).At present, LH has been widely used in the surgical treatment of a variety of benign and malignant liver diseases, and is expected to replace traditional open surgery, becoming the “gold standard” for surgical treatment of certain liver diseases (8). However, intraoperative hemorrhage and carbon dioxide gas embolization are still the biggest difficulties in LH (9, 10). Intraoperative hemorrhage not only increases the risk of complications caused by blood transfusion, but also relates to the high recurrence rate and low survival rate in patients with malignant tumors (11). Hemorrhage during LH includes hemorrhage from the Glisson system and hemorrhage from the hepatic venous system. Considering that the portal vein and hepatic artery branches of the Glisson system are thick, surrounded by Glisson sheaths, the wall is easily contracted and not easily torn after injury, and it is easier to clip and suture blood vessels by Pringle Maneuver to block the first hepatic portal blood flow, therefore, it is relatively simple to treat bleeding. It is well known that the hepatic venous system includes the left liver, the liver, the right hepatic vein and its branches, the short hepatic vein and the inferior vena cava. The difficulty in controlling hepatic venous hemorrhage is related to several reasons. First of all, the hepatic venous system has a large lumen, a thin wall, a large number of branching holes, and is easily damaged and torn. Second, the hepatic vein lacks a valve device that prevents blood reflux. Finally, the hepatic vein wall is not easily contracted in the liver parenchyma. In addition to the risk of major bleeding after injury, there is still a risk of CO2 gas embolism (12). Moreover,treating intraoperative bleeding in patients with tumors is even more difficult, since the vasculature at the tumor site is more permeable due to invasion of tumor cells (13). In the case of unintentional injury, tributaries of the hepatic veins may be the main source of bleeding. Prevention of venous injury remains a challenge, as the most suitable technique has not been established, but the risk of venous injury can be effectively reduced by accessing or exposing the anterior or posterior side of the hepatic vein prior to dissection of the lateral side (14). GE frequently occurs during laparoscopic hepatectomy, although most episodes of grade 1 embolism seem to be harmless (15). Severe CO2 embolization can quickly cause respiratory and circulatory dysfunction in patients, and if the treatment experience is not appropriate, it can cause serious consequences such as hypoxemia, heart failure, arrhythmia and even death. Laparoscopic surgery can be complicated by gas embolism, and although the incidence of this disease is estimated to be as low as 0.002% to 0.02%, the mortality rate is as high as 50%.The characteristic manifestation of gas embolism is cyanosis of the head and neck with a millwheel cardiac murmur due to obstruction of inflow to the right side of the heart. Signs of diagnosis include arrhythmia, hypoxia, and a sudden decrease in end-tidal carbon dioxide. However, the clinical manifestations of embolism in the subjects were not obvious. This also indicates the high risk of carbon dioxide embolism during laparoscopic surgery (16).

Therefore, prevention and control of hepatic venous system hemorrhage during LH and timely treatment of severe CO2 gas embolism caused by venous hemorrhage are the key to reducing perioperative complications of LH and promoting postoperative recovery. In the last two years, our center completed 60 cases of laparoscopic anatomical hepatectomy (type of hepatic segment resection as seen in **Table 1**, and clinical and surgical data in **Table 2**). This article will be based on the author’s institutional experience, combined with relevant literature to explore the hepatic venous system hemorrhage and CO2 gas embolization during the LH.

**Table 1:** Laparoscopic hepatectomy *via* hepatic vein approach(60 cases).

Resection of hepatic segment	Cases
II, III	13
II, III, IV	23
V, VI, VII, VIII	7
V, VIII	2
VI, VII	4
IV	2
VII	3
VIII	3
I	3
Total	60

**Table 2:** Clinical characteristics of 60 patients with laparoscopic hepatectomy.

Clinical characteristics	Value
Age	55.60 ± 14.35
Sex	
Male	22 (36.67%)
Female	38 (63.33%)
Operation time	150.17 ± 68.30
Intraoperative blood loss	230.59 ± 290.34
Postoperative hospital stay	7.49 ± 5.30
Major intraoperative events	
Suture of hepatic vein	5 (8.33%)
Carbon dioxide embolism	4 (6.67%)
Postoperative complications	
Bleeding	0 (0%)
Bile leakage	4 (6.67%)

## Materials and methods

### Patients and methods

This retrospective study was approved by the Institutional Review Board of Changzhou First People’s Hospital. All study participants gave written informed consent for the use of their clinical records. A total of 60 patients who received laparoscopic anatomical hepatectomy of hepatic vein approach for HCC from January 2019 to December 2020 at the Changzhou First People’s Hospital were retrospectively reviewed from our department’s prospective surgical database. A total of 60 patients underwent laparoscopic hepatectomy through the hepatic vein approach, according to the location of liver segment resection, there are 13 cases of II and III segments, 23 cases of II, III and IV, 7 cases of V, VI, VII and VIII, 2 cases of V and VIII, 4 cases of VI and VII, 2 cases of IV, 3 cases of VII, 3 cases of VIII and 3 cases of I. According to the disease type, there are 31 cases of hepatocellular carcinoma, 21 cases of intrahepatic bile duct stones, 3 cases of focal nodular hyperplasia, 3 cases of liver metastatic carcinoma, 1 case of hepatoblastoma, and 1 case of Inflammatory pseudotumor of the liver.

**Figure 1:**
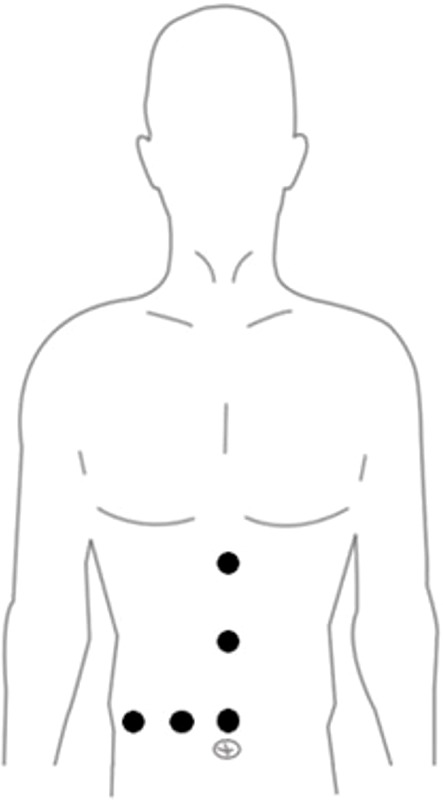
Port sites.

**Figure 2:**
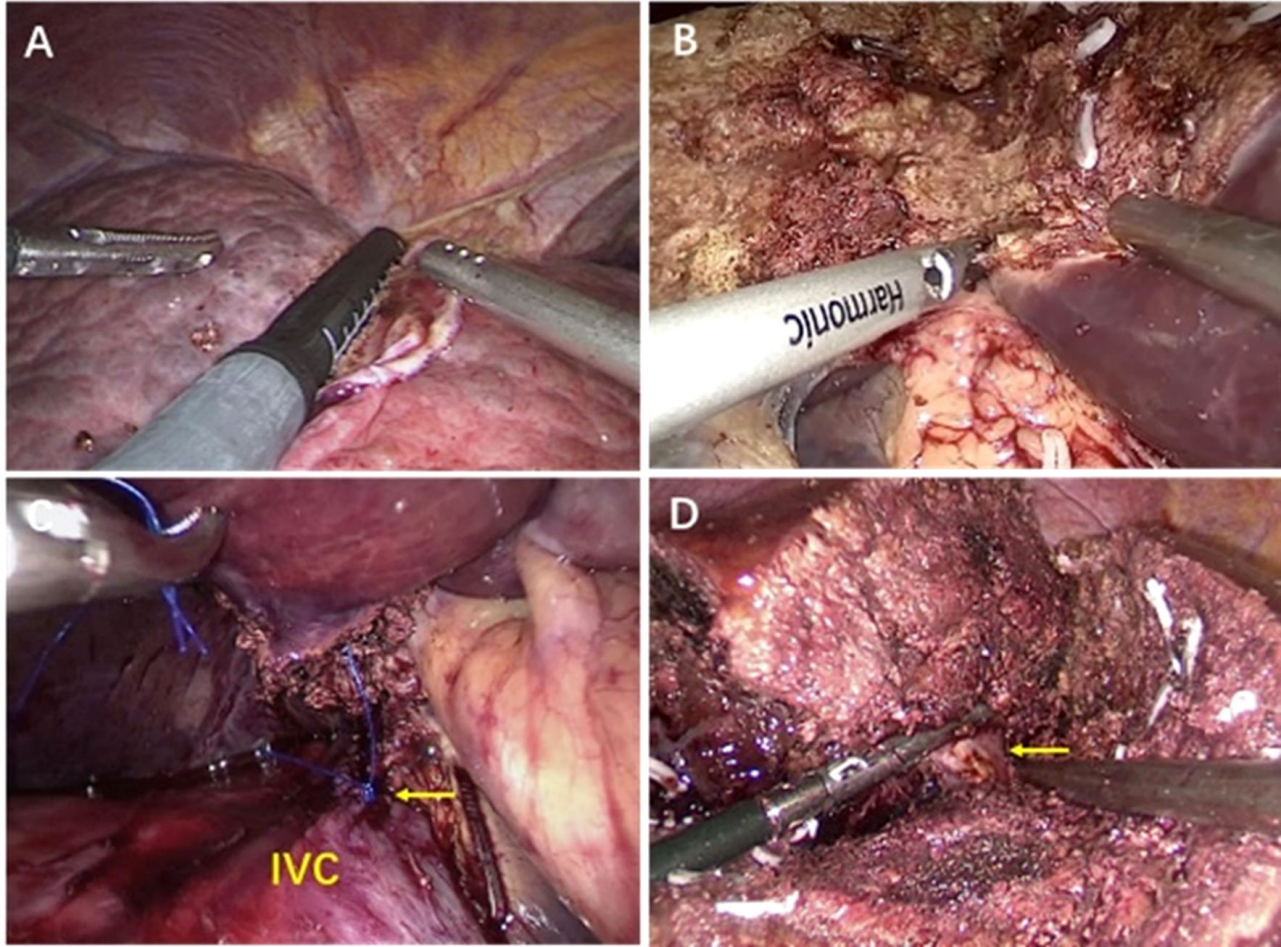
**(A)** By intraoperative ultrasound to localize liver tumors. **(B)** Transection of liver tissue with ultrasonic scalpel. **(C)** Used 5-0 prolene to suture laceration of retrohepatic inferior vena cava(RIVC) under laparoscope. **(D)** Laparoscopic separation of the VIII.

Surgical methods: After general anesthesia, the patient was placed in horizontal position, in which the right posterior lobe of the liver was excised with the right side raised and the right forearm fixed in the anesthesia frame.The Trocar has 4-6 Wells in total, and the location is fanned out according to the target liver segment. The observation and Pringle blocking holes are located around the umbilicus, and the main operating hole is placed on the extension line of the liver section (**Figure 1**). the intra-abdominal pressure was set to 12mmHg (1mmHg =0.133kPa), and the extrahepatic preset blocking band: the nylon band was pulled out of the blocking hole around the posterior two ends of the first hepatic hilum, and the plastic blocking tube was used to achieve repeated blocking and loosening *in vitro* in combination with the vascular clamp. The plane of the severed liver was obtained by intraoperative ultrasound localization of the main hepatic vein (**Figure 2A**), and the perihepatic bands were fully dissociated during right hemihepatectomy or right posterior lobe hepatectomy.During the operation, Pringle “15+5” mode (blocking the blood flow into the liver for 15min, releasing the blood flow for 5min, and repeating the circulation) was used to block the blood flow into the liver, and then the liver was broken.

The surgeon stood on the right side of the patient, held the ultrasonic knife in the right hand to cut the liver, and advanced from the tail side to the head side (**Figure 2B**). Among them, the ultrasonic knife was directly cut off the pipeline with diameter less than 1mm, the titanium clip was used to clamp the 1-2mm pipeline, and the Hem-O-LOCK was used to clamp the >2mm pipeline. The liver pedicle or hepatic vein root could also be cut off with a cutting closure device (**Figure 2D**).When the bile duct needed to be removed, the bile duct was dissected and closed. In the process of liver amputation, hemostasis was stopped by using an aspirator and a bipolar electrocoagulation section Surgicel compression hemostasis is used for the rupture of hepatic vein less than 3mm, a 4-0 prolene suture was used for hemostasis with the rupture>3mm (**Figure 2C**). The direction of intraoperative liver resection was from the foot to the head, from shallow to deep, and with the help of intraoperative ultrasound guidance, the main hepatic vein was gradually exposed. At the same time, intraoperative anesthesia should be combined with control of central venous pressure (CVP), and CVP <5cm H2O is safe for LH.

## Results

**Figure 3:**
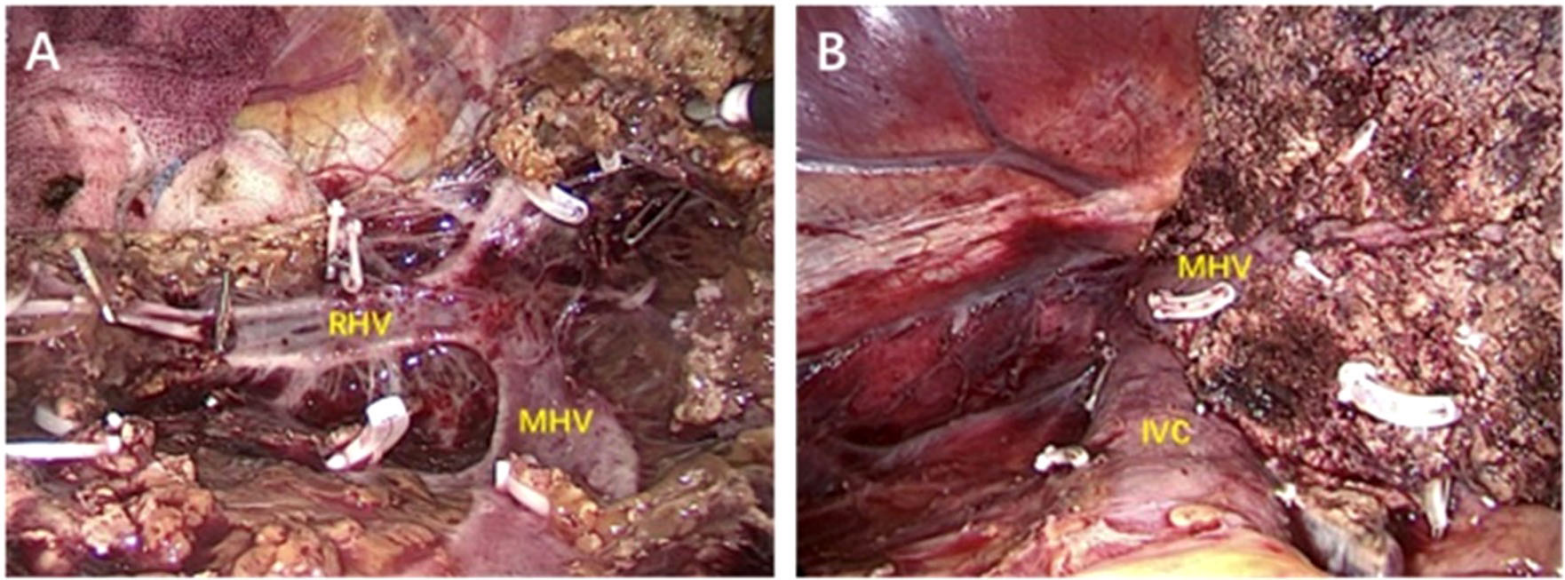
**(A)** Resection of the segment VIII, the right hepatic vein (RHV) and middle hepatic vein (MHV) were exposed during the operation. **(B)** After right lobe hepatectomy, the inferior vena cava (IVC) and middle hepatic vein (MHV) were exposed.

1. A total of 60 patients underwent laparoscopic hepatectomy *via* hepatic vein approach, and the specific surgical methods are shown in **Table 1**. The images of different segmentectomy are shown in **Figures 2**, **3**. During the operation, the left, middle or right hepatic veins were clearly exposed according to the needs of the target hepatic segment resection.

2. In this group, 36 cases were intrathecal occlusion and 24 cases were extrathecal occlusion, among which 51 cases were combined with Pringle (15 + 5min) to block the first hilar for a maximum of 5 times, and 22 cases were dissected with the second hilar combined with hepatic vein pre-occlusion during operation. The operation time was 150.17 ± 68.30 min, the intraoperative blood loss was 230.59 ± 290.34 mL, and there was no conversion to open surgery (**Table 2**).

3.Serious complications occurred in 13 cases.There were 5 cases of hepatic vein hemorrhage, and one of them was the rupture of cutting stapling device in the right hepatic vein root, and the nail used for fixing is loosened.The bleeding stopped after the vessel was sutured. 4 cases of carbon dioxide gas embolism were ameliorated by suspension of pneumoperitoneum, head high and foot low right elevation, positive end-expiratory pressure ventilation, and proper hemostasis of hepatic vein (**Table 2**). ALT and AST were (205.67 ± 223.04) U/L and (189.91 ± 194.04) U/L on the first day after operation, and (158.63 ± 153.90) U/L and (63.28 ± 64.63) U/L and (63.28 ± 64.63) U/L on the third day after operation.There were no cases of abdominal bleeding and 4 cases of bile leakage, which were cured by conservative treatment. No reoperation or operative death occurred.The postoperative hospital stay was 7.49 ± 5.30 days.

## Discussion

LH started relatively late, and it could only be performed in a few centers in the 1990s. However, with the technological innovation, the improvement of surgical techniques and the accumulation of surgeons’ experience, laparoscopic hepatectomy has made great progress in minimally invasive liver surgery in the late 21st century (17). Liver resection *via* hepatic vein approach was first advocated by Makuuchi in Japan, and has become one of the standards of anatomical liver resection. He proposed that the intrahepatic marker for anatomical hepatectomy is the hepatic vein,accurate anatomy and exposure of intraoperative hepatic vein is the key to laparoscopic anatomic hepatectomy through hepatic vein (18). According to a recent international literature review, LH is associated with less blood loss and lower postoperative morbidity compared with open hepatectomy, but does not differ significantly in terms of tumor outcome (19). The difficulty lies in the prevention and treatment of intraoperative hepatic vein bleeding, gas embolism and other complications. “Tenting sign of hepatic veins” is an important anatomical knowledge that can help us reduce bleeding during surgery (20). Previous studies have shown that keeping PP below 12mmhg may reduce the occurrence of carbon dioxide embolism (21).In this study, when intraoperative CO2 embolism occurred, pneumoperitoneum suspension, head high foot low right elevation, and positive end-expiratory pressure ventilation were used to rapidly improve the situation. After reading a large number of literatures, I conclude that the most important treatment for intraoperative CO2 embolization is to reduce pneumoperitoneum pressure, control CVP, and increase ventilation. Takechi K’s paper addressed CO2 embolism as follows: 1. Lower pneumoperitoneum pressure, but proceed with laparoscopic surgery. 2. Abandon the Pringle technique. 3. The fraction of inhaled oxygen was increased to 1.0, and intravenous phenylephrine (0.1 mg) was administered (22). But interestingly, intraoperative bleeding and carbon dioxide embolism seem to be the opposite of the coin. The Second International Consensus Conference on Laparoscopic Hepatectomy, based on 54 publications, made the following recommendations for intraoperative bleeding control: 1. Use a high pressure pneumoperitoneum of 10-14 MMHG. 2. Use a CVP of less than 5mmHg (23).Moreover,in order to improve the postoperative prognosis of patients and reduce the intraoperative risk, I think the following steps are essential.

### Preoperative accurate assessment

Most patients with laparoscopic hepatectomy in China have a background of hepatitis and cirrhosis, and have poor liver reserve and tolerance to ischemia-reperfusion injury, so they are more likely to bleed during liver resection. Accurate assessment of the patient’s liver function, the relationship between the lesion and important blood vessels before surgery is essential to prevent intraoperative hepatic venous bleeding. At present, it is mainly evaluated by ultrasound (**Figure 2A**), CT, MRI and other instruments to determine the extent of liver resection and the anatomical relationship of the main liver pipeline, especially the hepatic vein system, and then select the best surgical plan.

Three-dimensional virtual liver technology has been able to display intrahepatic vessels with a diameter of more than 1 mm, especially for the details of the intrahepatic vasculature (24). For large-scale liver resection, special site hepatectomy and other complex LH involving deep hepatic parenchyma, I recommend three-dimensional visual assessment, and carry out three-dimensional reconstruction based on two-dimensional imaging data to comprehensively evaluate the spatial stereo relationship between the lesion and the hepatic artery, portal vein, bile duct, hepatic vein or inferior vena cava (11). In addition, preoperative simulated segmental hepatectomy can be performed by using virtual surgical software to predict the major pipeline structures and complex important dangers that may be encountered during the actual operation of laparoscopic liver resection. It is necessary to cope with the dangerous parts that are prone to bleeding and the trunk and important branches of the hepatic vein that may be involved in order to effectively prevent the risk of hemorrhage of the hepatic venous system during operation.

### Methods to block blood flow in the hepatic vein system

Compared with traditional open liver resection, once hepatic venous system hemorrhage occurs during LH, the treatment is relatively more difficult (25). During the laparoscopic surgery, surgeons can not use the conventional methods such as top, pressure, pinch and other conditions under laparotomy to control bleeding, and it is difficult to complete the precise suture to stop bleeding in time, moreover, the bleeding will also contaminate the lens, affect the visual field, which further increase the difficulty of hemostasis. In order to reduce the risk of surgery and prevent hepatic venous system hemorrhage, appropriate hepatic blood flow blockage should be selected during surgery to avoid passive treatment after hemorrhage.

The first hepatic portal blood flow blockage can directly reduce the hepatic venous return blood volume, rapidly reduce the hepatic sinus, hepatic venous pressure and central venous pressure. In addition to effectively controlling the Glisson system bleeding, it is also effective in reducing hepatic venous system hemorrhage. Some scholars have pointed out that the anatomical separation of the second hepatic hilum may damage the hepatic vein, which may not only cause major bleeding, but also increase the incidence of CO2 gas embolism. Therefore, the suture of the liver parenchyma is more reliable than the separation of the second hepatic hilum outside the liver parenchyma (26). Blocking the third hepatic hilum should first fully dissipate the entire liver. The short hepatic vein and the right posterior inferior vein were separated from the bottom to the top and from the right to the left. The proximal end was clamped with an absorbable clip, and the distal end was ligated and disconnecte (27). Chen’s simple total hepatic blood flow blocking technique (Pringle method + lower hepatic inferior vena cava blockage) can significantly reduce the blood flow of the hepatic vein and the superior and inferior vena cava, rapidly reduce the pressure, and obtain a similar whole hepatic blood flow blockage. The clinical effect can safely and effectively control the hepatic section hemorrhage during operation, and the operation is simple and easy, which is conducive to clear and accurate liver resection (28).

### Application of various laparoscopic liver resection instruments

The removal of liver parenchyma in LH is inseparable from various instruments. The effective disconnection of hepatic veins and branches in liver parenchyma is one of the keys to prevent and control hepatic venous system hemorrhage. The author’s institution mainly uses the ultrasonic scalpel (**Figure 2B**), Cavitron Ultrasonic Surgical Aspirator (CUSA) and endoscopic GIA (Endo-GIA) for liver parenchyma resection.

Ultrasonic scalpel is mainly used to cut thin layer of liver tissue, can close the blood vessel with diameter <3mm without damage, while the blood vessel with diameter >3mm needs to be clamped with titanium clip (29). By oscillating to rupture of the liver cells, thereby retaining the intact structure of the blood vessels and bile ducts, the ultrasonic scalpel has the advantages of shortening the time of the liver parenchyma, low thermal damage, and less bleeding (30). The method of using the ultrasonic knife to cut off the liver is “small-mouth engulfment, layer-by-layer advancement”. When the liver tissue is condensed and cut, the cutter head is used for clamping, crushing, pushing and separating. The metal working surface of the cutter head should always be in the visible state, try to stay away from blood vessels, and do not blindly penetrate the liver parenchyma to cause blood vessels to burn.

CUSA is a versatile device that destroys and absorbs tissue cells with high water content, while highly elastic tissue with high collagen content (such as blood vessels and biliary system) is not destroyed, thus reducing the damage to normal tissues to the lowest (31). CUSA is especially suitable for deep liver parenchyma, which is conducive to fine dissection of the pipe structure and disconnection. The use of CUSA should select the appropriate power based on the pathology of the liver parenchyma. For the important pipeline structure on the section, especially the hepatic vein branch, it should be fully dissected and dissected to achieve full-dimensional nakedness. After confirming the diameter of the tube and walking, then the vessel clamp is properly clamped and then disconnected. It is forbidden to cut the blood vessel with an ultrasonic scalpel without fully exposing the blood vessel, or cut it with the blood vessel clamp clamping half of the blood vessel.

Endo-GIA staple cartridges can be divided into white nails, blue nails and golden nails according to the different nail heights. In the operation, different nail cartridges can be used to disconnect the traffic branches in the liver parenchyma according to the thickness of the liver tissue. Endo-GIA can disconnect liver tissue, blood vessels and biliary branches at one time, speeding up the operation and increasing the safety of surgery (32). (**Figure 2D**). For patients with partial vascular variability, in order to avoid accidental injury, laparoscopic ultrasound-assisted positioning can be used to determine the location of the vessel before the Endo-GIA is used to disconnect the vessel.

### Intraoperative fine operation to prevent hepatic venous system hemorrhage and CO2 gas embolism

Hepatic veins vary greatly in walking in the liver, making it difficult to find a fixed treatment pattern. The important pipeline structure that is difficult to confirm during operation should be carefully identified with the anatomical landmark of the liver surface and the ischemic boundary line after regional hepatic blood flow blockade. If necessary, combined with laparoscopic intraoperative ultrasound (**Figure 2A**), etc. to prevent accidental injury.

When dissecting the liver and treating the hepatic vein root and the short hepatic vein, the interstitial space should be identified, and the correct direction, angle and strength should be grasped. The operation should be gentle, avoid forced separation, and puncture the blood vessels. It has been reported that the second hepatic hilum is dissected and the corresponding hepatic vein is sutured through the liver before the liver is cut. We have also tried a few cases, and the effect of stopping bleeding during the operation is obvious. During LH, attention should be paid to maintaining a moderate tension in the section and cleaning the field. If the tension is too small, the section cannot be unfolded, affecting the visual field, exposure and operation; if the tension is too large, the hepatic vein branch of the section may be torn to cause bleeding or CO2 gas embolism.

Good vision is the premise of laparoscopic operation. During the operation, the surgical field should be kept clean and dry, avoiding smoke and blood. The lens holder should adjust the lens angle at any time to avoid collision between the lens and the operating instrument and the lens. The suction device adopts a point suction or flushing method to ensure sufficient pneumoperitoneal pressure and operation space while sucking up blood and smoke. Blind operation in the “blood pool” should be avoided in case of hepatic venous system hemorrhage, otherwise the hepatic vein trunk may be damaged, resulting in fatal bleeding and severe CO2 gas embolism.

### Controlled low central venous pressure technology

Low central venous pressure (LCVP) technology refers to reducing the pressure of hepatic sinuses and intravenous, reducing the pressure gradient inside and outside the blood vessel wall, thereby reducing the amount of bleeding in the process of liver substantive separation, and reducing postoperative blood transfusion, shortening hospital time, reducing postoperative complications, etc (33). However, in LH, the safety and feasibility of LCVP application is still controversial due to pneumoperitoneal pressure and the risk of potential CO2 gas embolism. Animal experiments by Jayaraman et al (12). showed that the incidence of air embolism was positively correlated with the ratio of pneumoperitoneum pressure and central venous pressure. When the ratio increased, the incidence of air embolism increased significantly. Therefore, the balance of pneumoperitoneum pressure and hepatic venous pressure is one of the key factors determining hepatic venous system hemorrhage during LH. When the pneumoperitoneum pressure is lower than the hepatic venous pressure, hepatic venous hemorrhage occurs. Otherwise, CO2 gas embolism occurs. Kobayashi S et al. ‘s experiment showed that the probability of pulmonary gas embolism increased when the central venous pressure was lower than the intra-abdominal pressure (34).

Reducing central venous pressure is mainly achieved by limiting the amount of infusion, diuresis, and expansion of peripheral blood vessels. In the liver resection, the use of head high and low feet supine position can reduce the blood flow of the lower extremity vein and peripheral vein, which is helpful for reducing central venous pressure and reducing bleeding. There is no uniform standard for reducing central venous pressure. Liu Zhe et al. (35)believe that during the process of hepatic parenchymal resection, as long as there is no large vein and its branch damage, it is relatively safe to control the central venous pressure at 0-5 cmH2O, which can reduce the bleeding during LH. It should be noted that due to the influence of pneumoperitoneal pressure and body position during LH, the value of central venous pressure monitored during surgery is generally inaccurate and can only be used as a reference. Comprehensive judgment should be made based on clinical features such as oozing blood in the operation section, degree of filling of the hepatic vein and inferior vena cava, and the opinions of the anesthesiologist.

### Treatment of hepatic venous system hemorrhage

If bleeding occurs accidentally during LH, the surgeons should keep calm, accurately and timely determine the source of the bleeding, and take appropriate measures to quickly and effectively stop bleeding. Hepatic venous system hemorrhage is characterized by a darker color and a “pulsed” gush. Hepatic vein accidental injury should immediately block the first hepatic portal blood flow into the liver. The left hand uses the device to temporarily compress the bleeding site to reduce bleeding. The assistant uses the aspirator to quickly and accurately remove the blood, clearly revealing the blood site and the degree of blood vessel damage. If it is a small vein branch bleeding in the liver section, bipolar electrocoagulation can be used to stop bleeding. For hepatic vein trunk or larger branch avulsion, rupture and hemorrhage, if the rupture is small, hemostatic can be stopped by partial compression such as gauze or hemostatic cotton. For hepatic vein trunk or larger branch avulsion, rupture and hemorrhage, if the rupture is small, hemostatic can be stopped by partial compression such as gauze or hemostatic cotton. If the rupture is slightly larger, after separating the liver tissue surrounding the damaged blood vessel and fully exposing the vein trunk and branches, clip the vessel at both ends of the rupture, or repair the ruptured vein with a 4-0 or 5-0 Prolene suture (**Figure 2C**).

It should be noted that after determining the bleeding site do not blindly clamp or largely stitch to stop the bleeding, should try to dissect the exposed blood vessel trend, accurately determine the source of bleeding, and its pipe diameter, walking, crack position, size, etc., and then choose the appropriate method to deal with. In general, most bleeding can be controlled and treated under laparoscopy. For the hepatic vein root and inferior vena cava laceration, it is recommended to quickly fill with gauze, and promptly turn to open the laparotomy.

### Treatment of severe CO2 gas embolism during operation

LH often involves important branches of the liver vein, and surgeons are mostly accustomed to using higher abdominal pressure and lower CVP to help reduce intraoperative bleeding, which makes CO2 easier to enter the cavity vein system, thereby increasing the incidence of CO2 embolism. Especially for laparoscopic hepatectomy, the cross-section is difficult to expose, the liver parenchyma is deep in the liver or the right hepatic vein branch, and the operation time is longer, which leads to a significant increase in the probability of severe CO2 gas embolism during surgery.

In our institution, there were 4 patients with typical CO2 gas embolism due to hepatic vein trunk or branch injury during laparoscopic liver resection (**Table 2**), which showed that arterial blood pressure dropped rapidly to 80/50 mmHg without bleeding or only a small amount of bleeding, the blood oxygen saturation dropped below 80%, and the end-expiratory C02 partial pressure (EtCO2) rapidly dropped below 25 mmHg. By suspending the operation, using gauze or hemostatic material to urgently compress and fill the venous breach, reducing the pneumoperitoneum pressure or changing to no pneumoperitoneum state, using the head low foot high position, changing the ventilation mode to end-expiratory positive pressure ventilation (PEEP), increasing the amount of fluid, increasing the central venous pressure, the abnormal indicators began to return to normal after about 3-10min. The treatment in the paper of Hou W et al. is as follows: pneumoperitoneus is stopped, Trendelenburg position that facilitates the flow of gas into the apex of the right ventricle and prevents it from entering the pulmonary artery (36) is adjusted, and air bubbles are released from the central line (37).In view of the serious consequences that CO2 gas embolism can cause, we believe that the possibility of CO2 gas embolism should be considered in patients with no significant hemorrhage during surgery but accompanied by sudden hemodynamic changes, or a decrease in EtCO2, or spO2. Among above, the early warning consciousness of the surgeon, the roving nurse and the anesthesiologist is the key to early detection of severe CO2 gas embolism. Second, TEE can quickly help diagnose embolism and determine the extent and location of embolism (37).

In short, with the deep understanding of liver imaging and anatomy, the improvement of laparoscopic technique, the accumulation of surgical experience and the continuous updating of surgical instruments, the treatment of hepatic venous system hemorrhage and severe CO2 gas embolism during LH operation will be further improved. However, in view of the tearing of the main trunk or important branches of the hepatic vein, it not only causes dangerous bleeding, but also may cause CO2 gas embolism to affect the body’s circulation which may lead to fatal complications. It is recommended that LH should be performed in larger medical centers by surgeons with experience of laparoscopic surgery.

## Data availability statement

The original contributions presented in the study are included in the article/supplementary material. Further inquiries can be directed to the corresponding authors.

## Ethics statement

The studies involving human participants were reviewed and approved by the Ethics Committee of the Third Affiliated Hospital of Soochow University. The patients/participants provided their written informed consent to participate in this study.

## Author contributions

Conception and design: ZQ, Y-FD, and X-ZH. Data collection: K-JW, J-WF, D-SS and JC. Writing the article: ZQ, K-JW, J-WF, D-SS, JC, Y-FD, Y-XC, and D-LS. Critical revision of the article: Y-FD, and X-ZH. Final approval of the article: ZQ, K-JW, D-SS, JC, Y-XC, D-LS, Y-FD, and X-ZH. Statistical analysis: ZQ, K-JW, D-SS and JC. Obtained funding: ZQ and Y-FD. Overall responsibility: Y-FD. All authors contributed to the article and approved the submitted version.
